# The effect of school meals with fatty fish on adolescents’ self-reported symptoms for mental health: FINS-TEENS - a randomized controlled intervention trial

**DOI:** 10.1080/16546628.2017.1383818

**Published:** 2017-10-12

**Authors:** Siv Skotheim, Katina Handeland, Marian Kjellevold, Jannike Øyen, Livar Frøyland, Øyvind Lie, Ingvild Eide Graff, Valborg Baste, Kjell Morten Stormark, Lisbeth Dahl

**Affiliations:** ^a^ Regional Centre for Child and Youth Mental Health and Child Welfare, Uni Research Health, Bergen, Norway; ^b^ National Institute of Nutrition and Seafood Research (NIFES), Bergen, Norway; ^c^ Department of Clinical medicine, Faculty of Medicine and Dentistry, University of Bergen, Bergen, Norway; ^d^ Uni Research Health, Bergen, Norway; ^e^ Department of Health Promotion and Development, University of Bergen, Bergen, Norway

**Keywords:** Dietary intervention, school meals, fatty fish, mental health, adolescence

## Abstract

There is a growing body of evidence linking fish consumption and n-3 LCPUFAs to mental health. Still, the results from randomized trials with n-3 LCPUFAs show conflicting results, and it is possible that the combined effect of several nutrients in fish may explain the observed associations. To aim of the present study was to investigate if school meals with fatty fish three times per week for 12 weeks could alter mental health in a sample of typically developing adolescents. In the Fish Intervention Studies-TEENS (FINS-TEENS), adolescents from eight secondary schools (n=425) in Norway, were randomized to receive school meals with fatty fish, meat or n-3 LCPUFA supplements. Mental health was assessed with the Strengths and Difficulties Questionnaire (SDQ) and the differences between the groups were assessed with linear mixed effect models, unadjusted and adjusted for baseline and dietary compliance. The results showed no effects of school meals with fatty fish compared to similar meals with meat or n-3 LCPUFAs on the adolescents’ self-reported symptom scores for mental health. Among adolescents scoring above the SDQ cut-offs (high-scorers), the fish- improved less than the meat group in the self-reported symptom scores for total difficulties- and emotional problems. However, the findings should be regarded as preliminary, as the analyses for the high-scorer group were underpowered. In conclusion, serving school meals with fatty fish did not alter mental health in a typically developing sample of adolescents. It is possible that serving healthy school meals with meat is more beneficial than similar meals with fatty fish in adolescents scoring high on mental health problems. However, the results should be seen as preliminary, as the dietary compliance in the fish group was low and the analyses in the high score group underpowered. Thus, further studies should investigate the associations between fish consumption and adolescents’ mental health.

## Introduction

Mental health disorders affect a significant proportion of children and adolescents. A recent meta-analysis found a worldwide prevalence of 13% of children and adolescents, with an anxiety disorder being the most prevalent (6.5%) [1]. In Norway, the prevalence is estimated to be around 7% in pre-[2] and primary [3] school children, and a large population-based survey found an increase in self-reported mental health problems during adolescence [4]. In addition, a substantial number report subthreshold symptoms that may lead to functional impairment [5].

An unhealthy dietary pattern has been associated with poor mental health in both adults [6,7], children and adolescents [8], even though the precise mechanisms are unclear [9]. A general concern is given to the effects of the typically modern Western dietary pattern, which is characterized by high levels of meat and saturated fat and low levels of fish and vegetables [10]. Fish is rich in n-3 long chain polyunsaturated fatty acids (n-3 LCPUFAs) and other important nutrients, such as vitamin D, selenium, iodine and high-quality protein [11,12]. Especially the n-3 LCPUFAs have received considerable interest, and there is growing evidence that suboptimal intakes of n-3 LCPUFAs may be associated with mental health over the lifespan [13,14]. The most prominent findings have been revealed for depression [15], but there is also some support for an effect of n-3 LCPUFAs in subgroups of children with attention-deficit hyperactivity disorder (ADHD) [16]. Still, findings from randomized controlled trials (RCT) with clinical populations remain inconclusive [14] and no benefits on mental health were seen in a sample of typically developing children after supplementation with n-3 LCPUFAs for 16 weeks [17]. However, long-term effects were found for both externalizing and internalizing problems, after 6 months supplementation with n-3 LCPUFAs in a community-residing sample of children (8–16 y), suggesting a delayed effect of n-3 LCPUFAs [18]. Recently, evidence has also accumulated for the impact of vitamin D and the overall results from cross-sectional and longitudinal studies suggest that vitamin D plays a role in the pathogenesis of mental health in both children and adolescents [19].

Most studies have focused on the effect of supplementing with single micro- or macro-nutrients and only a few studies have explored the relationship between consumption of fish as food and mental health. One study found an inverse relationship between fish consumption and major depression across countries [20] and two surveys from Finland found an association between infrequent fish intake and depression in women [21,22]. Longitudinal studies have also found a negative association between maternal intake of seafood during pregnancy and suboptimal child outcomes, such as IQ, social development and communication skills [23], as well as a negative association between the n-3 LCPUFAs, docosahexaenoic (DHA) status early in life and child internalizing problems (anxious/depressed) at 7 years of age [24]. However, to our knowledge no study has investigated the possible impact of an increased intake of fatty fish, rich on n-3 LCPUFAs and other important nutrients, on mental health status in a sample of typically developing adolescents.

The aim of the present study was to investigate whether lunch meals with fatty fish three times per week for 12 weeks altered mental health status compared to identical control meals with meat or supplements with n-3 LCPUFAs in a typically developing sample of adolescents.

## Subjects and methods

### Source population and participants

The source population in the Fish Intervention Studies-TEENS (FINS-TEENS) were adolescents attending 9th grade (14–15 years old) at eight secondary schools in Bergen, Norway. All the 26 secondary schools in the municipality were contacted. Three schools never replied, nine refused to participate and six were excluded because they had less than three school classes in 9th grade. Thus, eight secondary schools with 785 adolescents attending 9th grade were invited to take part in the study and written consent was obtained from 481 (61%) adolescents and one of their parents/caregivers. Exclusion criteria were allergy or intolerance to the food or supplements included in the intervention. The trial was conducted between February and May 2015 in accordance with the declaration of Helsinki. Ethical approval was obtained from the Norwegian Data Protection Official for Research (project number: 41030) and the trial is registered in ClinicalTrials.gov (NCT02350322).

### Trial

A three-armed randomized controlled study design was used. The participants received a school meal with either fatty fish (Fish group), a comparable meal with meat/cheese (Meat group) or fish oil supplements containing n-3 LCPUFAs (Supplement group). The meals and supplements were served to participants three times a week for 12 weeks in the classrooms during lunch break. The meals were prepared by a catering agency (Søtt+Salt A/S). The meals in the fish group consisted of salmon, mackerel and herring, whereas the meals in the meat group consisted of chicken, turkey and beefburger (sometimes cheese was served together with the meat). Halal meat was provided on request and pork meat was not used. All meals consisted of vegetables and/or salad in combination with mostly wholegrain pasta, focaccia, baguette or tortilla. The amount of fish and meat was requested to be 80–100 grams per meal, and thus 90 gram fatty fish per servings were used to calculate the weekly intake of n-3 LCPUFAs in the supplement group. Each capsule contained 500 milligrams (mg) of concentrated fish oil (Nycoplus® Omega-3, 500 mg produced by Takeda Nycomed, Asker, Norway) and eight capsules per serving corresponded to 90 gram of oily fish. Trained research assistants were responsible for handing out the meals/supplements in each classroom and monitored compliance by registering the amount of leftovers from every adolescent, on a scale ranging from 0 (none of the served food was consumed) to 4 (all of the served food was consumed). Likewise, the intake of supplements were counted and scored according to the number of capsules (0–7) consumed. Records of dietary compliance revealed that there were significant differences in intake between the three intervention groups, and that the proportion of participants who consumed at least half of the meals/capsules during the trial was 38%, 66% and 87% in the fish, meat and supplement group, respectively [25]. A more detailed description of the design, study meals and dietary compliance are given in Skotheim et al. [25].

### Procedure

The SDQ was administered to the participants at school as part of a larger computer-based questionnaire, including a food frequency questionnaire (FFQ) and questions about background characteristics. The participants had access to individual computers and the questionnaire was filled out before lunch break at both pre- and post-intervention. In addition, the participants took part in a concentration and reading- and spelling test, and biological samples were collected (not used in this present article). The same group of researchers was responsible for the data collection at pre- and post-intervention, and was present in the classroom to answer questions from the participants.

### Measurements

### Mental health

The SDQ is a brief screening questionnaire, measuring mental health during the last 6 months in youths between the age of 3–16 [26]. The instrument consists of 25 items divided on five subscales: Emotional symptoms, conduct problems, hyperactivity/inattention symptoms, peer relationship problems and prosocial behaviour. Each item is rated on a scale from 0–2, and it is possible to get a score on each subscales (0–10), as well as a total difficulties score based on the first four difficulties subscales (0–40). SDQ also includes an impact supplement, which assess the adolescent’s level of distress and interference of symptoms and problems on daily life functioning in youths reporting mental health problems. Moreover, a follow-up version of the SDQ has been developed in order to target any changes due to an intervention. The follow-up version is identical to the original SDQ, but asks about the last month as opposed to last 6 months, as in the original SDQ.

The SDQ may be completed by several informants (self, parent and teacher) and the present study used the self-completed (SDQ-S) version (11–16 y). The psychometric properties of the Norwegian version of SDQ-S has been investigated in a systematic review [27], that included 39 571 children. The analyses supported its construct validity and the internal consistency for the total difficulties scale and the subscale emotional problems were satisfactory, while the internal consistency for the reaming subscales were somewhat lower, especially for the subscale conduct problems.

Norwegian cut-off points for the SDQ-S were used to define the participants that scored high on the SDQ total difficulties or the five subscales (high scorer). The cut-offs were based on a survey, that included 4167 participants (11–16 years old) from Norway, where the 80th percentile were used to determine the participants that scores in the borderline or clinical range on the SDQ-S total difficulties or the five subscales [28]. Thus, high scorers were defined as those who scored ≥ 5 on emotional symptoms, ≥ 4 on conduct problems, ≥ 6 on hyperactivity/inattention symptoms, ≥ 4 on peer problems, or ≥ 15 on total difficulties. As the prosocial scale is inverted compared to the others subscales, ‘high scorers’ were defines as those scoring ≤ 5 on the scale. The impact supplement was administered but is not reported here.

### Dietary habits and background characteristics

A revised and extended version of a validated food frequency questionnaire (FFQ) [29,30] was completed by the adolescents both before and after the intervention to assess their habitual diet during the last 3 months. The purpose of the FFQ was to monitor the participants’ habitual diet (i.e. what participants ate besides the intervention meals and supplements). It was a semi-quantitative FFQ, comprising 34 questions, measuring the frequencies of consuming different groups (i.e. milk and dairy products, fruits and vegetables, etc.). In addition, the FFQ included questions related to physical activity and characteristics, such as age, gender, weight, height and ethnicity. A more detailed description of the FFQ used in the present study is given in Handeland et al. [31]. However, as previously shown, there were no changes in the participants’ habitual diet during the intervention period [32].

Body mass index (BMI) was calculated by dividing each adolescent’s weight (kilogram) by the square of the height (metres). In addition, Cole’s age and sex-specific BMI cut-off points for underweight [33] and overweight [34] according to adolescents (14.5 years) were used to define the proportions that where underweight, normal and overweight/obese. At post-intervention the adolescents were instructed to not include the meals/supplements served in the study [31]. In addition, one of the parents/caregivers received an email with a link to an online questionnaire at the same time points, measuring demographic factors, such as education, household income and marital status and the adolescents’ mental health status (SDQ-P).

### Randomization

Participants were individually randomized to one of the three groups, stratified by gender. Pieces of papers marked with one of the three intervention groups were put in two boxes: one marked ‘girls’ and one marked ‘boys’. Two researchers assigned every enrolled girl and boy, to either the fish or the meat or the supplement group by drawing lots. The researcher who drew lots was only informed about the participants’ gender (blinded), while the other researcher who had access to the list with the participants’ names and class affiliation, registered the assigned intervention for each individual in a spreadsheet.

### Sample size

This three-armed intervention study, had two repeated measurements (pre- and post-intervention) with an assumed correlation of 0.5. Sample size was calculated based on the primary outcome of the trial (d2 test of attention), where a small to moderate effect size (cohens *d *= 0.35) was applied. Given a power of 80% and a significance level of α = 0.05, it was estimated that a sample size of 119 participants in each group was needed. With the risk of 20% dropout, totally 446 participants ought to be enrolled.

### Statistical analyses

Continuous variables were expressed in means and standard deviation (SD). Categorical variables were expressed in numbers and percentages. Differences between the groups at baseline were assessed with a one-way ANOVA (continuous variables) or Chi-square test (categorical variables). Differences between completers (valid data pre- and post-intervention) and non-completers (withdrawn or missing SDQ data pre or post), were assessed with independent samples t-test or Chi-square test.

Paired-samples t-test was used to analyse differences between pre- and post-scores on the SDQ (total difficulties and the five subscales) within each intervention group. To investigate differences between groups in change SDQ scores (Δ SDQ scores), linear mixed effect models were applied. The participants’ school class was included as a random intercept to account for dependency in the data at the level of class affiliation. Two models were presented. In the first model, the currently examined SDQ outcome at post-intervention, was adjusted for the equivalent outcome at baseline. In the second model, dietary compliance was added to the model. The fish group was used as reference. Model assumptions were investigated by visual inspections of residual and normal probability plots. Possible interaction effects between group and compliance were investigated for all SDQ outcomes, but not shown because no significant effects were found. Two-tailed p-values < 0.05 were considered statistically significant.

Statistical analyses were carried out using the Statistical Package for the Social Sciences (SPSS® Statistics version 24, IBM Corporation, US) and Stata Statistical Software (STATA/IC 14.2).

## Results

### Subjects

**Figure 1.**
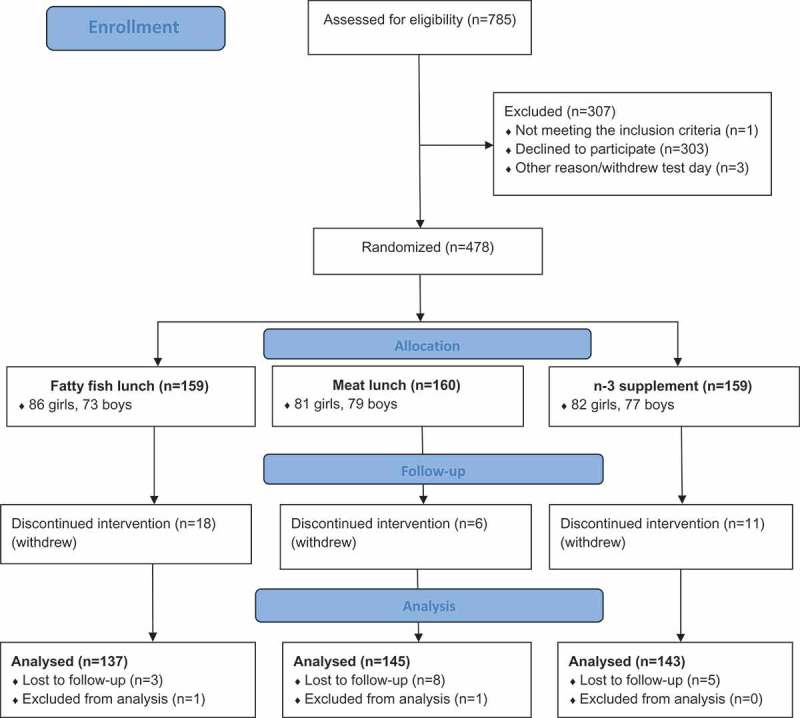
Flow chart over participants.

Out of the 481 adolescents who agreed to participate in the trial, three withdrew the day of baseline testing and before randomization. Thus, 478 adolescents were randomized to one of the three treatment groups. During the intervention, 34 pupils withdrew (actively) from the study and 16 were lost to follow-up during the administration of SDQ, either pre or post. In addition, one participant withdrew his consent after study completion and two unusual/extreme change scores on the outcome variable of interest (ΔSDQ Total > 30, scale 0–40, mean change score = -0.12, SD = 4.1) were identified through inspection of boxplot and excluded from the analyses (interpreted as non-valid responses). Thus, the present study included 425 participants (Figure 1).

There were no differences between the completers and non-completers (withdrawn and lost to follow-up) in any of the baseline characteristics. Regarding the SDQ, the non-completers scored higher on emotional problems, hyperactivity/inattention and total difficulties than the completers at baseline. However, there were no differences on any of the SDQ scales between the three intervention groups neither for the non-completer or the completer sample at baseline (data not shown).

### Characteristics of the study population

No differences in baseline characteristics were found between the three intervention groups (Table 1). The participants had a mean age of 14.6 years and 53% were girls. About 79% of the participants had a BMI within the normal range, while 14% was defined as underweight and 7% as overweight/obese. Approximately 11% had a non-Norwegian background. About 71% of the mothers and 59% of the fathers had higher education, and 28% reported a high household income. On average, the participants consumed seafood for dinner once per week and about 17% reported taken n-3 LCPUFAs supplements on a daily basis during the last 3 months.

**Table 1.** Baseline characteristics of the study population and the different intervention groups.

	n	All	Fish (n = 137)	Meat (n = 145)	Supplement*^a^* (n = 143)
Gender, girls, n (%)	425	224 (53)	77 (56)	72 (50)	75 (52)
Age, mean (SD)	425	14.6 (.34)	14.6 (.34)	14.6 (.33)	14.6 (.34)
BMI category ^b^	396				
Underweight		55 (14)	21 (17)	19 (14)	15 (11)
Normal weight		312 (79)	98 (77)	105 (78)	109 (81)
Overweight/obese		29 (7)	8 (6)	10 (8)	11 (8)
Ethnicity, non-Norwegian^c^, n (%)	425	46 (11)	16 (12)	16 (11)	14 (10)
Maternal education level, n (%)	346				
Elementary/high or vocational school		101 (29)	26 (24)	40 (35)	35 (29)
College/University		246 (71)	84 (76)	75 (65)	87 (60)
Paternal education level, n (%)	345				
Elementary/high or vocational school		142 (41)	51 (46)	47 (41)	44 (37)
College/University		204 (59)	59 (54)	68 (59)	77 (64)
Household income, n (%)	345				
< 200.000–749.999		73 (21)	21 (19)	20 (18)	32 (26)
750.000–1. 249 999		175 (51)	57 (52)	62 (54)	56 (46)
1 250 000- >2.000 000		97 (28)	32 (29)	32 (28)	33 (27)
Seafood for dinner^d^, mean (SD)	425	4.1 (.95)	4.1 (.99)	4.0 (.94)	4.1 (.90)
Omega-3 supplement, n (99%)	424				
Never		229 (54)	67 (49)	81 (56)	81 (57)
1–3 times/month		53 (13)	18 (13)	18 (12)	17 (12)
1–3 times/week		47 (11)	21 (15)	14 (10)	12 (8)
4–6 times/week		22 (5)	7 (5)	6 (4)	9 (6)
Daily		73 (17)	24 (18)	26 (18)	23 (16)

### Effects of the intervention on the adolescents’ self-reported SDQ scores

There were no differences in any of the SDQ change scores from pre to post between the fish and meat or fish and supplement group, neither before nor after adjusting for the participants’ baseline scores or baseline and compliance scores (Table 2).

**Table 2.** Predicted changes in SDQ scores after fish (n = 137), meat (n = 145) or supplement (n = 143) intervention, different models.

	Crude SDQ scores			SDQ scores adjusted for:			
				Baseline score		Baseline score & compliance	
SDQ scales	Baseline Mean (SD)	12 weeks Mean (SD)	*p-within^a^*	Change Mean (95% CI)	*p-value^b^*	Change Mean (95% CI)	*p-value^c^*
**Emotional problems**							
Fish	2.9 ± 2.1	2.9 ± 2.4	0.96	0.07 (-0.18,0.31)	-	0.12 (-0.15,0.39)	-
Meat	2.6 ± 2.1	2.6 ± 2.0	0.77	0.03 (-0.21,0.27)	0.83	0.02 (-0.22,0.26)	0.61
Supplement	2.5 ± 2.2	2.6 ± 2.1	0.47	0.05 (-0.19,0.29)	0.92	0.01 (-0.26,0.26)	0.56
**Conduct problems**							
Fish	1.6 ± 1.4	1.5 ± 1.4	0.74	−0.07 (-0.27,0.14)	-	−0.08 (-0.30,0.14)	-
Meat	1.7 ± 1.7	1.4 ± 1.4	0.01	−0.27 (-.047,-0.07)	0.13	−0.27 (-0.47,-0.07)	0.19
Supplement	1.6 ± 1.5	1.7 ± 1.5	0.26	0.10 (-0.10,0.30)	0.23	0.11 (-0.10,0.33)	0.23
**Hyperactivity/in attention**							
Fish	3.9 ± 2.3	3.8 ± 2.2	0.41	−0.10 (-0.34,0.16)	-	−0.17 (-0.44,0.11)	-
Meat	3.9 ± 2.2	4.0 ± 2.2	0.58	0.10 (-0.15, 0.35)	0.28	0.11 (-0.14,0.35)	0.14
Supplement	3.6 ± 2.1	3.6 ± 2.1	0.88	−0.08 (-0.32,0.17)	0.92	−0.01 (-0.27,0.26)	0.44
**Peer problems**							
Fish	1.5 ± 1.4	1.5 ± 1.4	0.88	−0.02 (-.22,0.17)	-	−0.03 (-0.24,0.18)	-
Meat	1.7 ± 1.6	1.5 ± 1.5	0.09	−0.16 (-0.35, 0.03)	0.31	−0.16 (-0.35,0.03)	0.37
Supplement	1.4 ± 1.5	1.5 ± 1.4	0.80	−0.02 (-0.21,0.17)	0.99	−0.01 (-0.22,0.19)	0.93
**Prosocial behaviour**							
Fish	7.6 ± 1.8	7.5 ± 1.7	0.91	−0.02 (-0.25,0.22)	-	0.10 (-0.16, 0.36)	-
Meat	7.6 ± 1.7	7.5 ± 1.8	0.79	−0.03 (-0.26,0.19)	0.93	−0.04 (-0.27,0.19)	0.42
Supplement	7.6 ± 1.7	7.6 ± 1.8	0.79	0.04 (-0.19,0.26)	0.75	−0.07 (-0.31,0.18)	0.39
**Total difficulties**							
Fish	9.9 ± 4.9	9.7 ± 5.1	0.52	−0.11 (-0.65,0.44)	-	−0.12 (-0.72,0.48)	-
Meat	9.9 ± 5.3	9.6 ± 4.6	0.23	−0.33 (-0.90,0.20)	0.57	−0.32 (-0.85,0.21)	0.62
Supplement	9.2 ± 4.9	9.4 ± 5.1	0.47	0.08 (-0.45,0.62)	0.63	0.10 (-0.48,0.67)	0.63

### Effects of the intervention on high SDQ-scorers at baseline

In the high-scorer sample, the fish group improved less in the symptom scores for emotional problems (*p *= 0.04) and total difficulties (*p* = 0.02) than the meat group. The difference remained significant for emotional problems (*p* = 0.03), and was borderline significant for total difficulties (*p* = 0.06) after adjusting for compliance (Table 3).

**Table 3.** Predicted changes in SDQ scores for participants with high SDQ scores at baseline after fish, meat or supplement intervention, different models.

		Crude SDQ scores			SDQ scores adjusted for:			
					Baseline score		Baseline score & compliance	
SDQ scales	n	Baseline Mean (SD)	12 weeks Mean (SD)	*p-within^a^*	Change Mean (95% CI)	*p-value^b^*	Change Mean (95% CI)	*p-value^c^*
**Emotional problems (≥ 5)**								
Fish	26	6.4 ± 1.3	6.0 ± 1.9	0.25	−0.31 (-0.92,0.30)	-	−0.21 (-0.89,0.47)	-
Meat	31	5.8 ± 1.1	4.7 ± 1.5	<0.01	−1.20 (-1.75,-0.64)	0.04	−1.20 (-1.76,-0.63)	0.03
Supplement	29	6.1 ± 1.4	5.2 ± 1.9	<0.01	−0.82 (-1.39,-0.25)	0.23	−0.91 (-0.89,0.47)	0.17
**Conduct problems (≥ 4)**								
Fish	13	4.5 ± 1.1	2.9 ± 1.9	<0.01	−1.64 (-2.39,-0.89)	-	−1,38 (-2,23,-0,48)	-
Meat	19	5.1 ± 1.4	3.5 ± 1.5	<0.01	−1.53 (-2.16,-0.91)	0.83	−1,56 (-2,19,-0.93)	0.75
Supplement	17	4.5 ± 0.6	3.5 ± 1.5	<0.01	−0.98 (-1.63,-0.32)	0.18	−1,14 (-1,87,-0.41)	0.71
**Hyperactivity/inattention (≥ 6)**								
Fish	33	7.2 ± 1.3	6.2 ± 1.5	<0.01	−0.90 (-1.44,-0.37)	-	−0.88 (-1.50,-0.26)	-
Meat	31	7.1 ± 1.1	6.6 ± 1.4	0.07	−0.44 (-0.99,0.11)	0.23	−0.44 (-1.00,0.12)	0.28
Supplement	29	6.8 ± 1.0	5.6 ± 2.1	<0.01	−1.32 (-1.89,-0.75)	0.29	−1.35 (-2,0,-0.66)	0.37
**Peer problems(≥ 4)**								
Fish	15	4.3 ± 0.5	2.9 ± 1.1	<0.01	−1.47 (-2.28,-0.65)	-	−1.26 (-2.15,-0.38)	-
Meat	18	4.8 ± 1.2	3.1 ± 1.9	<0.01	−1.95 (-2.70,-1.20)	0.78	−1.95 (-2.69,-1.22)	0.72
Supplement	11	5.0 ± 1.0	3.6 ± 1.2	0.03	−1.31 (-2.23,-0.39)	0.32	−1.26 (-2.15,-0.38)	0.20
**Prosocial behaviour (≤ 5)**								
Fish	20	4.6 ± 1.0	5.6 ± 1.4	<0.01	0.84 (0.15,1.52)	-	0.88 (0.15,1.61)	-
Meat	19	4.4 ± 1.2	5.5 ± 1.9	0.02	1.05 (0.35,1.75)	0.63	1.06 (0.35,1.77)	0.69
Supplement	21	4.7 ± 0.6	5.5 ± 1.3	0.02	0.76 (1.0, 1.42)	0.86	0.7 (-0.02,1.45)	0.75
**Total difficulties (≥15)**								
Fish	25	17.8 ± 2.9	16.4 ± 3.9	0.02	−1.54 (-3.01,0–08)	-	−1.88 (-3.60,-0.15)	-
Meat	26	18.6 ± 3.5	14.3 ± 3.6	<0.01	−4.11 (-5.55,-2.67)	0.02	−4.10 (-5.54,-2.65)	0.06
Supplement	20	18.0 ± 2.6	15.3 ± 4.7	<0.01	−2.78 (-4.42,-1.14)	0.27	−2.38 (-4.34,-0.42)	0.73

## Discussion

The overall results from the present study revealed that being served fatty fish three times a week for 12 weeks did not alter the adolescents’ self-reported symptom scores for mental health, compared to being served comparable meals with meat or n-3 LCPUFA supplements. In the high SDQ scorer sample the fish improved less than the meat group on the symptom scores for total difficulties and emotional problems, and the findings remained significant for emotional problems and borderline significant for total difficulties after adjusting for compliance.

To our knowledge, no RCTs have previously assessed the effect of a dietary intervention with fatty fish on mental health in a sample of typically developing adolescents. However, the lack of findings for both the fatty fish and the meat group, are in concordance with the results from a large cluster randomized intervention trial, showing no reduction in the risk of being in the borderline/abnormal range on any of the SDQ dimensions after a 3 months intervention with school breakfast as rated by the teacher (primary school) or self-report (secondary school) [35]. The findings are also consistent with the results from a randomized intervention trial supplementing with n-3 LCPUFAs or placebo for 16 weeks [17]. In this study, no beneficial effects of n-3 LCPUFAs on mental health scores as measured by the SDQ (parent and teacher reports) were found in 8–10 year old children from a mainstream school population. On the contrary, long-term reductions were found for both externalizing and internalizing behaviour problems in an RCT supplementing with n-3 LCPUFAs for 6 months in a community-residing sample of children and adolescents (8–16 y) [18]. Interestingly, the strongest effects were found 6 months after the end of treatment, suggesting that the accumulation of fatty acids in the brain may take some time before changes can be seen on mental health. Taken together the results indicate that there are no immediate effects on mental health after short-term intervention with either healthy school meals or n-3 LCPUFA supplementation in typically developing children and adolescents. Cross-sectional and prospective studies show that fish consumption is associated with reduced levels of mental health problems in both children and adults [20–24], thus a longer exposure time than 3 months is possibly required in order to influence mental health. Given the low dietary compliance in the fatty fish group compared to the two other intervention groups, it is not certain whether an extension of time in the present study would have yielded a sufficient intake of fatty fish. As we were not able to serve warm lunch meals, future intervention trials with fatty fish should possibly give priority to creating warm meals that highly match adolescents’ preferences in order to ensure a higher dietary compliance than achieved in the present study. It is also possible that other research designs are better for investigating the relationship between fatty fish and mental health in a normal sample of adolescents, such as prospective studies over a longer period of time that include validated dietary assessments and that adjust for important confounders [8,36].

The finding that the adolescents with higher levels of emotional problems and total difficulties (emotional problems included here) benefitted more from receiving healthy lunch meals with meat compared to comparable meals with fatty fish, could indicate that micronutrients typically found in meat, such as iron, contributed to a positive change. However, mostly white meat (chicken and turkey) were served in the present study and it is unlikely that iron from the intervention contributed to the present finding. As the dietary compliance in the fish group was low compared to the meat group [25], a possible explanation is therefore that a higher consumption of lunch meals with better nutritional composition than their habitual packed lunch was beneficial for the adolescents with emotional problems. As already reported, the participants’ habitual diet was below the recommendations for fish, fruits and vegetables [31], which support this interpretation. In addition, a recent systematic review found evidence for a consistent trend between good quality diet and lower levels of internalizing problems (low mood and anxiety) in children and adolescents, and some evidence for the reverse [8]. However, as this finding was based on a smaller sub-sample that participated in the intervention, the findings should be replicated on a larger sample of adolescents with high levels of emotional problems.

One of the most important limitations with the present trial was the low dietary compliance in the fish group compared to the two other intervention groups. Thus, the intake of fatty fish might not have been sufficient to influence mental health. A potential limitation is also the duration of 12 weeks, which might have been too short to alter mental health status. It was also a limitation that that we did not have a second follow-up. The turn-over of fatty acids in the brain is likely to be slower in older children than during the last trimester of pregnancy and the first month after birth, and it is possible that the impact of an increased intake of fatty fish and n-3 LCPUFAs first reveals itself after some time than right after the exposure [18]. The generalizability of the results is also weakened by the fact that 40% of the adolescents from the participating schools refused to participate in the study. Even though we managed to recruit schools from various socio-economic districts of Bergen [25], it is a problem that those who refuse to participate in research often are different from those who participate on important variables, such as socio-economic status, mental health problems and nutritional habits [38,39]. The proportion with a university/college education among the parents/caregivers in the present sample was higher than the general population: between 30-59 years in Norway [40]. In addition, there was a systematic difference between the completers and non-completers with respect to the SDQ symptoms scores, indicating that those with higher mental health problems and who probably would have profited most from the intervention were under-represented in the present study. Moreover, the findings shown for the high SDQ scorer sample should be regarded as preliminary, as the analyses conducted for this sub-sample most likely were underpowered. Strengths in the present study were that the adolescents were randomized independently of class affiliation, reducing the potential bias caused by clusters of data and not the intervention. An important strength is also that we kept detailed registrations of actual intake (dietary compliance) throughout the trial. As dietary compliance might be more demanding when intervening with food instead of supplements, the registrations of dietary compliance made it possible to include this as a covariate in the analyses.

## Conclusion

In summary, no beneficial effect of an increased intake of fatty fish on mental health as measured with the SDQ were found, in a sample of typically developing adolescents or a high SDQ scorer sample from Norway. Thus, whether there is any causality between fatty fish consumption and mental health in adolescents still remains unestablished. Therefore, further research should investigate the associations between a healthy diet and fish consumption on adolescents’ mental health status. This can be carried out either with a prospective study emphasizing validated dietary intake and a longer follow-up period or with an RCT design, prioritizing the serving of warm, tasty meals to ensure acceptable compliance, preferably also in a group of adolescents with suboptimal nutritional status at baseline.

## Clinical trial registry number and website

Norwegian Data Protection Official for Research project number: 41030. ClinicalTrials.gov registration number: NCT02350322
